# UAV and Satellite Synergies for Mapping Grassland Aboveground Biomass in Hulunbuir Meadow Steppe

**DOI:** 10.3390/plants13071006

**Published:** 2024-03-31

**Authors:** Xiaohua Zhu, Xinyu Chen, Lingling Ma, Wei Liu

**Affiliations:** 1 National Engineering Laboratory for Satellite Remote Sensing Applications, Aerospace Information Research Institute, Chinese Academy of Sciences, Beijing 100094, China; chenxinyu221@mails.ucas.ac.cn (X.C.); mall@aircas.ac.cn (L.M.); 2 College of Resources and Environment, University of Chinese Academy of Sciences, Beijing 101408, China; 3 State Key Laboratory of Vegetation and Environmental Change, Institute of Botany, Chinese Academy of Sciences, Beijing 100093, China; lw076@ibcas.ac.cn

**Keywords:** aboveground biomass, uncrewed aerial vehicle, GF-2 multi-spectral data, random forest, leaf area index

## Abstract

Aboveground biomass (AGB) is an important indicator of the grassland ecosystem. It can be used to evaluate the grassland productivity and carbon stock. Satellite remote sensing technology is useful for monitoring the dynamic changes in AGB across a wide range of grasslands. However, due to the scale mismatch between satellite observations and ground surveys, significant uncertainties and biases exist in mapping grassland AGB from satellite data. This is also a common problem in low- and medium-resolution satellite remote sensing modeling that has not been effectively solved. The rapid development of uncrewed aerial vehicle (UAV) technology offers a way to solve this problem. In this study, we developed a method with UAV and satellite synergies for estimating grassland AGB that filled the gap between satellite observation and ground surveys and successfully mapped the grassland AGB in the Hulunbuir meadow steppe in the northeast of Inner Mongolia, China. First, based on the UAV hyperspectral data and ground survey data, the UAV-based AGB was estimated using a combination of typical vegetation indices (VIs) and the leaf area index (LAI), a structural parameter. Then, the UAV-based AGB was aggregated as a satellite-scale sample set and used to model satellite-based AGB estimation. At the same time, spatial information was incorporated into the LAI inversion process to minimize the scale bias between UAV and satellite data. Finally, the grassland AGB of the entire experimental area was mapped and analyzed. The results show the following: (1) random forest (RF) had the best performance compared with simple regression (SR), partial least squares regression (PLSR) and back-propagation neural network (BPNN) for UAV-based AGB estimation, with an R^2^ of 0.80 and an RMSE of 76.03 g/m^2^. (2) Grassland AGB estimation through introducing LAI achieved higher accuracy. For UAV-based AGB estimation, the R^2^ was improved by an average of 10% and the RMSE was reduced by an average of 9%. For satellite-based AGB estimation, the R^2^ was increased from 0.70 to 0.75 and the RMSE was decreased from 78.24 g/m^2^ to 72.36 g/m^2^. (3) Based on sample aggregated UAV-based AGB and an LAI map, the accuracy of satellite-based AGB estimation was significantly improved. The R^2^ was increased from 0.57 to 0.75, and the RMSE was decreased from 99.38 g/m^2^ to 72.36 g/m^2^. This suggests that UAVs can bridge the gap between satellite observations and field measurements by providing a sufficient training dataset for model development and AGB estimation from satellite data.

## 1. Introduction

The grassland ecosystem is one of the important terrestrial ecosystems and plays an indispensable role in livestock forage supply, climate regulation, soil protection and the global carbon cycle [1,2,3]. Grassland degradation caused by overgrazing has become an economic and environmental problem in pastoral areas, including China [4], Brazil [5], etc. Grassland aboveground biomass (AGB), which is a crucial indicator of grassland sensitivity to climate change and human activity, serves as a significant entry point for studying the issue. Quantitative AGB estimation of grassland at a large scale is crucial for monitoring the changes in grassland ecology and production function over short and long periods of time.

AGB estimation is commonly achieved by using field measurements or remote sensing-based modeling. The former is destructive and time-consuming, and it is applicable only to small-scale monitoring [6], whereas remote sensing is non-destructive and provides a cost-effective method to monitor and map AGB frequently over large areas. Vegetation indices (VIs), calculated based on target information of spectral signals, can be used as the model parameter in place of the full spectral band for model construction, representing an important method for AGB estimation from remote sensing data [7,8]. The widely used normalized difference vegetation index (NDVI) is an effective and reliable indicator for studying the growth status and biomass of different kinds of vegetation [8,9,10,11]. In addition to the NDVI, other VIs have been used, including the relative vegetation index (RVI) [12], the relative difference vegetation index (RDVI) [13], the optimized soil-adjusted vegetation index (OSAVI) [14], the modified soil-adjusted vegetation index (MSAVI) [15] and the chlorophyll index (CI) [16]. However, most previous studies involved statistical models based on a single VI, and each has its own limitations and uncertainties [17].

Uncrewed aerial vehicle (UAV) imaging is increasingly being used as an emerging technology in the field of grasslands due to its mobility [18,19,20]. UAVs are also relatively inexpensive compared to other high-resolution remote sensing platforms and are compatible with a variety of sensors [20,21,22,23]. For example, high-resolution grassland images can be obtained by UAVs equipped with consumer-grade hyperspectral sensors [7]. UAVs can reduce the impact of clouds on imaging data when flying at low altitudes, giving them greater application advantages over high-resolution satellite platforms. Therefore, UAVs have been widely used for estimating grassland vegetation parameters, including plant height, biomass, leaf area index and chlorophyll content [4,7,24]. Pecina et al. [25] proposed an approach for mapping coastal meadow AGB based on a random forest algorithm and vegetation indices calculated from UAV data. Zhang et al. [26] proposed a non-destructive method for rapid acquisition of grassland AGB using UAV RGB images. Their study also indicated that the grassland AGB retrieved from UAV data could be used as sample datasets for satellite modeling. It is an effective method for estimating grassland AGB by constructing the relationships between field-measured AGB and remotely sensed data [27]. However, the accuracy of grassland AGB estimation models based on spectral information may only yield errors due to the phenomenon of different objects possessing the same spectra. More information needs to be mined and introduced for AGB modeling [28], including characteristic vegetation indices and vegetation structural parameters [20]. Leaf area index (LAI), an important vegetation structural parameter, is also a common indicator for AGB modeling [29,30]. Meanwhile, the inversion of LAI from remote sensing data has become increasingly mature, and its accuracy is guaranteed [31,32,33]. UAV technology captures vegetation spectral or structural information with high spatial and spectral resolution, enabling accurate estimation of vegetation biomass [20,23,34]. However, due to the limitation of UAVs’ endurance conditions and the processing costs of ultra-high-resolution data, the application of UAVs in large-scale grassland AGB estimation is still limited.

Satellite remote sensing technology has the advantage of high-frequency and large-range observation, which provides an important data source for grassland AGB monitoring in large areas. However, satellite-based models for estimating AGB are always affected by the amount of in situ data and the spatial heterogeneity of the measurements [35,36,37,38]. For example, the grassland AGB on the Qinghai–Tibet Plateau was estimated from MODIS data by matching pixels of 500 m resolution to small quadrats of 0.5 m × 0.5 m and 1 m × 1 m [36]. Compared with that of other natural ecosystems, the grassland AGB is more sensitive to environmental change, and its spatial distribution shows significant heterogeneity [39,40]. Therefore, it is more important to obtain as many ground observation data as possible to reduce the impact of surface spatial heterogeneity. Due to the complexity of the natural grassland ecological environment, the heavy work of ground survey and the damaging effects of surface sampling, it is difficult to collect intensive AGB field samples over large-scale grassland. A large number of destructive ground surveys may also increase the risk of grassland desertification. This limits the accuracy of grassland biomass estimation using satellite data because the accuracy of biomass inversion is closely related to the quality and quantity of the field sample dataset, and it increases with the number of field samples [41]. High-resolution satellite data, including QuickBird, WorldView, SkySat and GF, can solve the scale mismatch in this case. Because these satellites have a resolution of less than 1 m, they are relatively easily matched with the ground measurements for modeling a more precise and robust model [42,43]. However, the high cost of these satellite data cannot support large-scale grassland AGB estimations for dynamically assessing grassland ecosystem productivity.

Ground survey, UAV observation and satellite observation have their own advantages and disadvantages. UAV technology, as an intermediate platform, is useful for bridging the gaps between ground survey and traditional space-based platforms [44]. In terms of biomass estimation in the process of grassland degradation, grassland degradation resulted in a decrease in dominant species and grassland coverage, leading to a significant increase in surface spatial heterogeneity, which creates more uncertainty in grassland AGB estimation based on satellite data. UAV technology can effectively solve the problem by capturing fine-scale vegetation information. However, current research still mainly focuses on the use of UAV data to provide auxiliary data required for satellite inversion, including sample datasets [36,43] and subpixel land cover [45]. Many studies fail to realize the unification of satellite and UAV data, and they do not consider the possible problems in the coordination of satellite and UAV data, such as scale effects.

Therefore, considering the mentioned advantages and limitations of different platforms, a more feasible grassland biomass inversion method can be explored by constructing a ground–UAV–satellite integrated grassland AGB estimation model and introducing the LAI to solve the problem of missing structural parameters in the model. Scale effect is also an unavoidable problem in the joint application of multi-scale data [46,47]. However, the bias between multi-scale AGB retrieval from different platforms’ data has still not been fully analyzed and evaluated, especially when satellite data and UAV data are used cooperatively for biomass estimation of degraded grassland [43]. The scale bias increases the uncertainty of inversion. However, if the scale information can be extracted and used to optimize the inversion process, the inversion uncertainty caused by multi-scale data can be reduced and the inversion accuracy can be improved [46,48].

Thus, this study collected field-measured data of the grassland AGB and LAI and remote sensing data in the form of UAV hyperspectral data and Chinese GaoFen-2 (GF-2) multi-spectral data, developing a grassland AGB estimation method based on a combination of satellite, UAV and ground. This method corrects the scale problem in low- and medium-resolution satellite remote sensing modeling and provides a new strategy for estimating grassland biomass with high precision in a large range. The research questions of this study are as follows: (1) what is the applicability of several common regression models for grassland AGB estimation? These models are simple regression (SR), partial least squares regression (PLSR), back-propagation neural network (BPNN) and random forest (RF). (2) How much can the accuracy of grassland AGB estimation be improved after adding structural parameter LAI? (3) How can the scale effect be reduced in mapping grassland AGB based on integrated UAV and satellite data? (4) What are the impacts of grassland restoration measures such as fertilization and mowing on grassland AGB?

## 2. Methods and Materials

### 2.1. Study Area

To evaluate the effect of degraded grassland restoration and management, a comprehensive field campaign was carried out in the Hulunbuir degraded grassland restoration technology research and development platform (hereinafter referred to as the Experimental Platform, EP) during 20–26 July 2022. The Experimental Platform (central coordinates: 49°17′59.25″ N, 120°0′16.2″ E), located in the center of the Hulunbuir meadow steppe in the northeast of Inner Mongolia, China, was one of the most typical temperate meadow steppes in China, with a mean annual temperature of −2–1 °C and mean annual precipitation of 380–400 mm [49,50].

**Figure 1:**
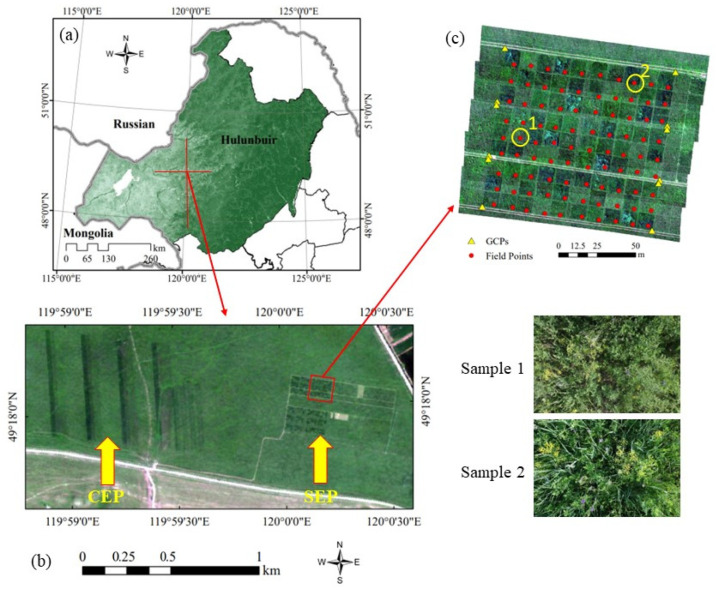
The location of the study area and the distribution of field measurements. (**a**) The study area is located in the center of the Hulunbuir meadow steppe in the northeast of Inner Mongolia, China; (**b**) Chinese GF-2 satellite image of the Experimental Platform. The platform was composed of a “strip” experimental platform (SEP) and a “cell” experimental platform (CEP); (**c**) UAV RGB image with 90 field-measured sample plots. Sample 1 and sample 2 are on-site photographs.

As shown in Figure 1, the Experimental Platform was composed of a “strip” experimental platform (SEP) and a “cell” experimental platform (CEP). Four groups of multi-year fertilization comparison quadrats were constructed in the “strip” test platform. The “cell” test platform developed a total of 1800 experimental units, each with an area of 10 m × 10 m. Fertilization control experiments had been carried out in 90 of these experimental units, which had formed different species composition and biomass gradients. Therefore, it was very suitable for grassland biomass estimation and change research. More than 30 dominant species were recorded in the Experimental Platform, including Poaceae (*Leymus chinensis*, *Stipa baicalensis* and *Cleistogenes squarrosa*), Fabaceae (*Astragalus laxmannii* and *Oxytropis myriophylla*), Asteraceae (*Artemisia scoparia*, *Artemisia frigida* and *Klasea centauroides*), Amaryllidaceae (*Allium tenuissimum* and *Allium polyrhizum*) and Ranunculaceae (*Thalictrum squarrosum* and *Clematis hexapetala*) [50,51]. According to field measurements, the average canopy height of the grassland was about 50 cm.

### 2.2. Data Acquisition

#### 2.2.1. Field Measurements

The field measurements were collected during 21–26 July 2022 in CEP. In total, 90 sample plots were set up, each with a size of 1 m × 1 m (elemental sampling unit, ESU), and 12 GCPs were placed for georeferencing. SVC HR-1024 (Spectra Vista, New York, NY, USA) was used during the campaign for measuring the surface reflectance of vegetation canopy, vegetation leaf and soil. Grassland LAI was collected using an LAI-2200 manufactured by LI-COR, Lincoln, NE, USA. The optical sensor of the LAI-2200 consisted of a fisheye lens (vertical field of view 148°, horizontal field of view 360°, wavelength range 320–490 nm) and an optical system. The fisheye lens “sees” a hemispherical image, which the optical system focuses onto the photodiode optical sensor [52]. The LAI-2200 infers LAI based on the Beer–Lambert law, which describes the relationship between the gap fraction and LAI [53]. Five concentric rings with a centered VZA of 7°, 23°, 38°, 53° and 68° were used in the LAI-2200 to determine the ratio of below-canopy to above-canopy measurements for calculating the gap fraction. According to the operation manual [52] and characteristic of grassland canopy, LAI measurements using the LAI-2200 were carried out with one above-canopy measurement and four below-canopy measurements of the incoming radiation in each sample plot. Such operations were repeated three times at a single point, and the average value was taken as the LAI value of the sample point. The average LAI was obtained based on one above-canopy measurement and four below-canopy measurements of the incoming radiation in each sample plot. The average LAI of each plot was calculated based on one above-canopy measurement and four low-canopy measurements. After LAI measurements, the plants in each ESU were harvested and dried in the laboratory. The AGB of each ESU was obtained by weighing the dried plants.

#### 2.2.2. UAV Hyperspectral Data Acquisition and Preprocessing

During the field campaign, a Nano-HP hyperspectral camera (Headwall, Boston, MA, USA) was equipped onto the DJI M300 UAV platform (DJI, Shenzhen, China) and used to obtain the grassland hyperspectral data over the Experimental Platform. Headwall’s Nano-Hp hyperspectral camera collects 270 spectral bands with 640 spatial elements within the visible-to-near-infrared (VNIR) range from 400 nm to 1000 nm, with a spectral resolution of approximately 2.2 nm. The pixel size of Nano-Hp hyperspectral camera is 7.4 μm.

The flight was conducted from 10:30 a.m. to 2:30 p.m. on 20 July 2022, and the weather was clear and cloudless. The UAV flew at a speed of 3 m/s and a height of 50 m above the ground, providing data at about 0.05 m resolution originally (focal length of 8 mm). Five images were collected during the UAV flight. A preprocessing series was carried out for UAV images, including radiometric correction, geometric correction, atmospheric correction and image splicing. Therefore, the information on geometric control points and the spectrum of reference target (calibration panel) was also obtained during flight. The data were processed in Headwall SpectralView software (Version 3.1), and a hyperspectral digital orthophoto map (DOM) was generated at 0.1 m resolution after processing. Then, the 12 field geometric control points (GCPs) were processed for georeferencing of the hyperspectral data to the reference system WGS 84 UTM 50 N. Atmospheric correction was carried out based on an empirical line approach. The reflectance of hyperspectral DOM was validated using in situ measurements, with uncertainty of approximately 2–3%.

#### 2.2.3. Chinese GF-2 Multi-Spectral Data Acquisition and Preprocessing

The Chinese GF-2 multi-spectral data covering the study area on 21 July 2022 were collected from the China Center for Resources Satellite Data and Application (CRESDA). GF-2 multi-spectral data had four bands (Table 1), the wavelength ranges of which were 450–520 nm, 520–590 nm, 630–690 nm and 770–890 nm. The resolution of the GF-2 multi-spectral data was 4 m. The preprocessing of the GF-2 multi-spectral data contained radiance calibration, atmospheric correction and geometric correction. Based on the band-specific absolute calibration gains, the at-satellite radiances (G_λ_) were calculated from 8-bit digital numbers (DNs) of GF-2 data. Then, the atmospheric correction was performed using a MODTRAN-based FLAASH (fast line-of-sight atmospheric analysis of spectral hypercubes) method. The reflectance was finally validated using in situ measurements, with uncertainty of approximately 4–8%. The geometric correction was performed using an image-to-image registration method based on previous geometrical corrected GF-2 data. The GF-2 image was registered in UTM zone 50 with a WGS84 datum.

### 2.3. Regression Models and Accuracy Assessment

Regression modeling based on machine learning has been increasingly applied to AGB inversion and proved to have reasonable validity [28,36]. As the typical machine learning regression methods, PLSR [54], RF [55] and BPNN [56] were analyzed in this study to establish the relationships between AGB and multi-source variables. RF is the machine learning regression method with the most applications in grassland AGB estimation, and the second is PLSR [41]. BPNN is a typical artificial neural network algorithm. At the same time, to evaluate the machine learning regression model, the simple regression method SR was also used for comparative analysis.

During AGB estimation, 60% of the field data were used for model training and the remaining 40% were used for model performance testing (20%) and accuracy evaluation (20%) of the above four regression models. The correlation coefficient (R^2^) and root-mean-square error (RMSE) of predicted AGB and measured AGB were calculated to assess the performance of different methods. R^2^ and RMSE can be calculated as follows:

(1) $$R^{2}=\frac{\sum_{i}^{n}\left({AGB}_{cal,i}-{AGB}_{cal}^{\prime })({AGB}_{obs,i}-{AGB}_{obs}^{\prime }\right)}{\sum_{i}^{n}{\left({AGB}_{cal,i}-{AGB}_{cal,i}^{\prime }\right)}^{2}\sum_{i}^{n}{\left({AGB}_{obs}-{AGB}_{obs}^{\prime }\right)}^{2}}$$

(2) $$RMSE=\sqrt{\frac{\sum_{i}^{n}{\left({AGB}_{cal,\;i}-{AGB}_{obs,i}\right)}^{2}}{n}}$$

where *n* is the number of samples, ${AGB}_{cal}$ is the predicted AGB, ${AGB}_{obs}$ is the measured AGB and ${AGB}_{obs}^{\prime }$ is the average value of measured AGB.

### 2.4. Estimation of Grassland Aboveground Biomass

**Figure 2:**
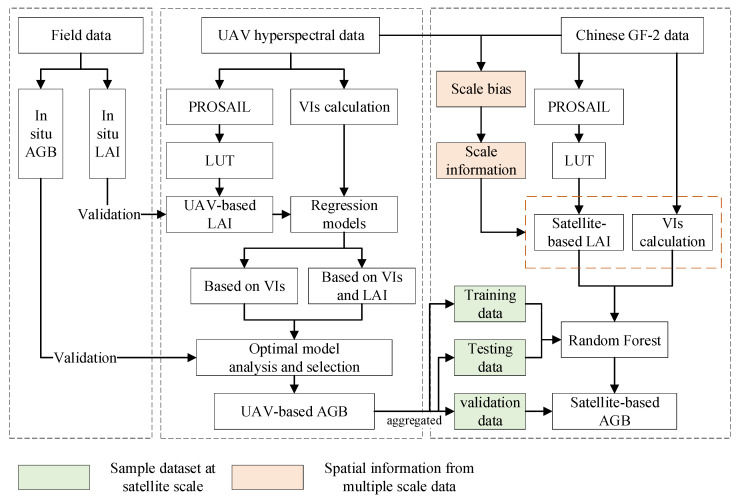
The flow of estimation of grassland aboveground biomass. Firstly, the field measurements were used for modeling and validation of AGB estimation from UAV data. The comparison between models, including SR, PLSR, BPNN and RF, was carried out with and without the vegetation structure parameter LAI. Then, the scale bias between UAV data and satellite data was extracted and used as prior information to correct the satellite-retrieved indicators. The UAV-based AGB was aggregated as the sample dataset for satellite remote sensing modeling. Finally, the satellite-based AGB was mapped and validated.

As shown in Figure 2, field data were used for estimation of the UAV-based AGB, and the best model was selected by comparing multiple models with and without the vegetation structure parameter LAI. Then, the UAV-based AGB was aggregated (spatial resolution is 4 m, consistent with the satellite resolution) as the sample dataset for satellite remote sensing modeling. Considering the strong correlation between the AGB and LAI, in the process of satellite LAI inversion, the scale bias between satellite data and UAV data was used as prior information to correct the satellite-retrieved LAI. The modified LAI was used as the key input for the satellite-based AGB inversion. Finally, the satellite-based AGB was mapped and validated against field data.

#### 2.4.1. Mapping UAV-Scale AGB from Hyperspectral Data

##### 2.4.1.1. Optimal VI Selection Based on UAV Hyperspectral Data

Six typical vegetation indices (VIs) were calculated and analyzed from UAV hyperspectral data for AGB estimation, as shown in Table 2, including the simple ratio vegetation index (RVI) and normalized difference vegetation index (NDVI). The 6 used VIs were proven to be well correlated with the grassland AGB [18,57]. To effectively use the UAV hyperspectral information, the correlation coefficient (R) values between the VIs created by combining any two spectral bands of UAV hyperspectral data and in situ AGB were calculated. The indicator R was drawn in the form of correlation plots and the most sensitive band combinations were selected with the maximum R.

##### 2.4.1.2. Generation of the LAI from UAV Hyperspectral Data Based on PROSAIL Model

First, the parameter sensitivity analysis and parameterization of the PROSAIL model [64,65,66] were carried out. For more details, refer to [7,67]. Then, a dataset with 100,000 simulations was generated by running PROSAIL in forward mode. Based on the simulated data and UAV hyperspectral data, the sensitive bands and two typical VIs (NDVI and OSAVI) were calculated to generate a look-up table (LUT). NDVI and OSAVI have been proved to be well correlated with the LAI [14,68]. Finally, the grassland LAI inversion based on the LUT was made by minimizing the RMSE between UAV reflectance data and reflectance found in the LUT.

##### 2.4.1.3. Estimation of the AGB from Hyperspectral Data

Based on the 6 VIs and LAI generated in Section 2.4.1.1 and Section 2.4.1.2, the UAV-based AGB was calculated in two cases.

Case 1. Estimation of the AGB based on VIs: the six VIs were used for retrieving and evaluating the grassland AGB from UAV hyperspectral data based on regression models, namely, simple regression (SR), partial least squares regression (PLSR), back-propagation neural network (BPNN) and random forest (RF).

Case 2. Estimation of AGB VIs and LAI: both VIs and the LAI were used for retrieving and evaluating the grassland AGB from UAV hyperspectral data based on the four regression models used in Case 1. Based on the estimated results, the impact of introducing the LAI on the estimation accuracy of AGB was evaluated.

The accuracy of the estimated results was verified using the in situ data. Based on the validation and evaluation of the different models, the model with the highest accuracy was selected as the optimal model for estimating the AGB from the Chinese GF-2 multi-spectral data.

#### 2.4.2. Mapping Satellite-Scale AGB from Chinese GF-2 Multi-Spectral Data

##### 2.4.2.1. Retrieval of the LAI Using PROSAIL and Multiple Spatial Information

First, based on the simulated data described in Section 2.4.1.2 and the spectral response function (SRF) of the Chinese GF-2 PMS sensor, the simulated bands and two typical VIs of NDVI and OSAVI were calculated to generate the satellite-based LUT. Due to the scale mismatch and spatial heterogeneity, there was an inversion bias when mapping the grassland LAI from satellite data [69]. Then, a spatial effect factor *k* was constructed based on Taylor development, which was used as scale information between the UAV-scale data and satellite-scale data for correcting the LAI inversion bias. The spatial effect factor k can be calculated with the following equation; for more details, refer to [46,68]. The spatial effect factor *k* is defined as:

(3) k=LAIp−LAIm

where *LAIp* is the LAI retrieved from observation data aggregated from high-resolution images; *LAIm* is the average LAI retrieved from high-resolution images, which is taken as a relative true value; and *k* is the difference between *LAIp* and *LAIm*, describing the bias between the true LAI value and the retrieved LAI value.

Because the LUT is not a normal function and the parameter LAI is sensitive to the NDVI, the purpose here was to obtain the scale information of different-resolution remote sensing data; thus, a semi-empirical formula was established for LAI calculation and finally used for spatial effect factor calculation.

(4) $$LAI=g\left(NDVI\right)=g(\frac{NIR-R}{NIR+R})=f\left(NIR,R\right)$$

where *NIR* and *R* are the near-infrared red band and the red band.

(5) $$k=f\left({NIR}_{m},R_{m}\right)-\frac{1}{n}\sum_{i=1}^{n}f\left({NIR}_{i},R_{i}\right)$$

where *NIR_i_* and *R_i_* are the reflectance of the near-infrared red band and the red band of high-resolution data; *NIR_m_* and Rm are the average of *NIR* and *R* and *n* is the number of pixels for aggregation. Then, $LAIm=\frac{1}{n}\sum_{i=1}^{n}f\left({NIR}_{i},R_{i}\right)$ is approximated using a second-order Taylor development of *f* around (*NIR_i_* = *NIR_m_*, *R_i_* = *R_m_*). Finally, the spatial effect factor *k* is calculated with the formulas shown below [46,69,70,71].

(6) $$\begin{array}{cc} k & =f(NIR_{m},R_{m})-\frac{1}{n}\sum_{i=1}^{n}\left[\begin{array}{l} f(NIR_{m},R_{m})+\left(\begin{array}{cc} \frac{\partial f}{\partial NIR} & \frac{\partial f}{\partial R} \end{array}\right)\left(\begin{array}{c} NIR_{i}-NIR_{m}\\ R_{i}-R_{m} \end{array}\right)\\ +\frac{1}{2}\left(\begin{array}{c} \begin{array}{cc} \frac{\partial^{2}f}{\partial NIR\partial NIR} & \frac{\partial^{2}f}{\partial R\partial NIR} \end{array}\\ \begin{array}{cc} \frac{\partial^{2}f}{\partial NIR\partial R} & \frac{\partial^{2}f}{\partial R\partial R} \end{array} \end{array}\right)\left(\begin{array}{c} \begin{array}{cc} {\left(NIR_{i}-NIR_{m}\right)}^{2} & \left(NIR_{i}-NIR_{m})(R_{i}-R_{m}\right) \end{array}\\ \begin{array}{cc} \left(R_{i}-R_{m})(NIR_{i}-NIR_{m}\right) & {\left(R_{i}-R_{m}\right)}^{2} \end{array} \end{array}\right) \end{array}\right]\\ & \;=-\frac{1}{2}\times \left(\frac{\partial^{2}f}{\partial NIR\partial NIR}\times VAR_{NIR}+\frac{\partial^{2}f}{\partial R\partial R}\times VAR_{R}+2\times \frac{\partial^{2}f}{\partial NIR\partial R}\times COV_{NIR,R}\right) \end{array}$$

where $\frac{\partial^{2}f}{\partial NIR\partial NIR}$, $\frac{\partial^{2}f}{\partial R\partial R}$ and $\frac{\partial^{2}f}{\partial NIR\partial R}$ are the second differential of function f(NIR,R); $VAR_{NIR}$ and $VAR_{R}$ are the variance of *NIR* and *R* and $COV_{NIR,R}$ is the covariance. Therefore, the difference between the LAIs retrieved from different resolution data is calculated using the variance of *NIR* and *R* value based on UAV-scale data and the second order differential of f(NIR,R) [46,68]. For the pixel of GF-2 without synchronized UAV flight data, a 40 × 40 window is used to calculate the variance and covariance for the current pixel.

Finally, a modified cost function is established based on spatial effect factor *k*.

(7) $$\delta =\sqrt{\frac{1}{n}\sum_{i}^{n}\frac{{\left(\rho_{i}^{o}-\rho_{i}^{s}\right)}^{2}}{\epsilon_{i}}+\frac{{\left(LAI-k-{LAI}^{e}\right)}^{2}}{\epsilon_{LAI}}}$$

where δ is the improved cost function based on scale information; $\rho_{i}^{o}$ is the spectral information of GF-2; $\rho_{i}^{s}$ is the simulated spectral information; $\epsilon_{i}\;$ is the error of measured reflectance; *k* is the scale effect factor; ${LAI}^{e}\;$ is the expectation value of LAI and $\epsilon_{LAI}$ is the error of measured LAI.

##### 2.4.2.2. Estimation of the AGB from GF-2 Multi-Spectral Data

By aggregating the UAV-scale AGB map, an AGB map with 4 m resolution (consistent with GF-2 multi-spectral data resolution) was obtained, which was called the satellite-scale sample set. All pixel values in the 4 m resolution AGB image were used as the sample data for satellite remote sensing modeling except null data and invalid data, making a total of 1057 samples. Of the 1057 samples, 60% were used for modeling, 20% for testing and 20% for validation. We developed satellite-scale AGB estimation models based on VIs (RVI, NDVI, RDVI, OSAVI and MSAVI) with and without LAI (described in Section 2.4.2.1) derived from the Chinese GF-2 multi-spectral data. The scale effect of VIs calculated from GF-2 data, also modified using Taylor series expansion based on the optimal model with lowest RMSE and highest R^2^, was selected as the satellite-based model. Finally, the grassland AGB in EP was mapped with the satellite-based model.

## 3. Results

### 3.1. Estimation of Grassland AGB from Hyperspectral Data

#### 3.1.1. AGB Estimation Based on VIs

**Figure 3:**
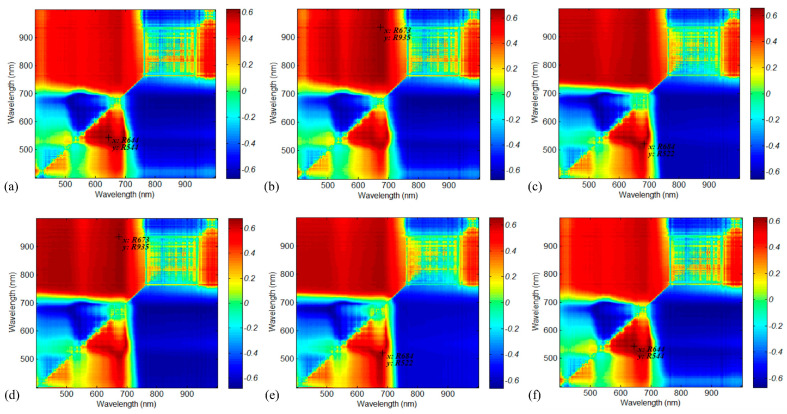
The correlation matrix between grassland AGB and VIs calculated from different spectral band combinations: (**a**) RVI, (**b**) NDVI, (**c**) RDVI, (**d**) OSAVI, (**e**) MSAVI, (**f**) CI. The six Vis of RVI_(644,544)_, NDVI_(673,935)_, RDVI_(684,522)_, OSAVI_(673,935)_, MSAVI_(684,522)_ and CI_(644,544)_ were selected for AGB estimation, with *R* = 0.63, 0.67, 0.65, 0.68, 0.66 and 0.63.

**Figure 4:**
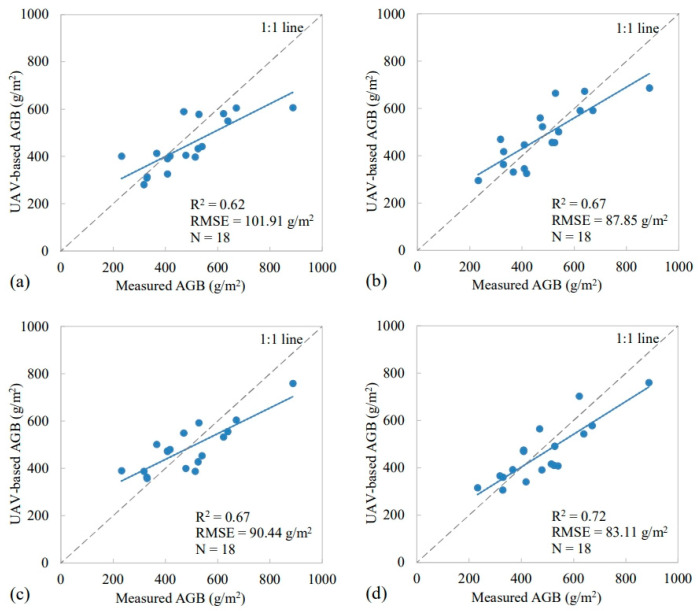
Scatterplot of UAV-based estimations versus ground measurements based on the methods of: (**a**) MLR, (**b**) PLSR, (**c**) BPNN, (**d**) RF. The blue line indicates the best fit, whereas the gray line indicates the 1:1 relationship.

As described in Section 2.4.1.1, considering the sensitivity of spectral information to biomass, the six VIs were calculated with any two spectral bands and ordered using the correlation coefficient *R*. Finally, the six VIs of RVI_(644,544)_, NDVI_(673,935)_, RDVI_(684,522)_, OSAVI_(673,935)_, MSAVI_(684,522)_ and CI_(644,544)_ were selected for AGB estimation, with *R* = 0.63, 0.67, 0.65, 0.68, 0.66 and 0.63. The correlation matrix is shown in Figure 3. Based on the selected VIs and regression models mentioned in Section 2.2, the UAV-based AGB was estimated and validated against the field-measured AGB (Figure 4). Among the regression models built with the selected VIs, the RF-based model achieved the best performance, with the R^2^ = 0.72 and the RMSE = 83.11 g/m^2^. The random forest’s training efficiency is high and can reduce overfitting problems; therefore, it was used as the main model for later inversion modeling.

#### 3.1.2. AGB Estimation Based on VIs and LAI

**Figure 5:**
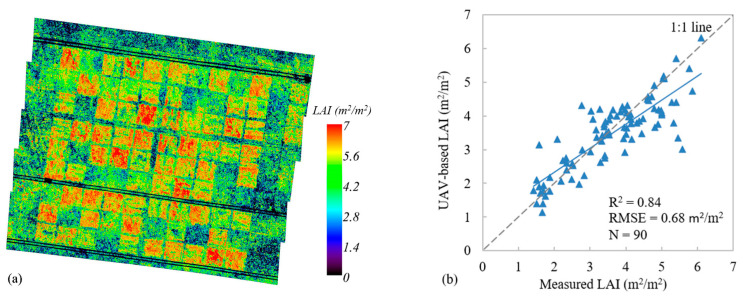
LAI estimation and validation. (**a**) The UAV-based LAI, (**b**) scatterplot of UAV-based LAI versus ground measurements. The blue line indicates the best fit, whereas the gray line indicates the 1:1 relationship.

Firstly, five bands with the highest correlation were selected based on the correlation analysis between the LAI and reflectance of each band. Then, two typical vegetation indices, NDVI and OSAVI, were also calculated using UAV hyperspectral data to construct the LUT for UAV-based LAI estimation. The UAV-based LAI was validated against the field-measured LAI (Figure 5b), with the R^2^ = 0.84 and the RMSE = 0.68 m^2^/m^2^. Finally, the UAV-based LAI would be used as vegetation structure information to participate in the UAV-based AGB mapping. As shown in Figure 4, since spectral information was prone to saturation in areas covered by dense vegetation, the AGB was underestimated. Therefore, the addition of LAI structure information can better improve the inversion accuracy of AGB. Compared with the AGB estimation model based on VIs only, the performance of AGB estimation model based on VIs+LAI was much better (Table 3). For the AGB estimation model based on RF, the R^2^ was increased from 0.72 to 0.80 and the RMSE was decreased from 83.11 m^2^/m^2^ to 76.03 m^2^/m^2^. The models constructed using RF with and without the LAI were both better than the others; thus, the regression model based on RF was selected as the final model for satellite-based AGB estimation.

### 3.2. Estimation of Grassland AGB from Chinese GF-2 Data

#### 3.2.1. AGB Estimation Based on VIs

Considering the lack of red-edge wavelengths, only five wideband VIs, namely, RVI, NDVI, RDVI, OSAVI and MSAVI, were calculated from the Chinese GF-2 multi-spectral data. Based on the five VIs and satellite-scale AGB sample set (aggregated from the UAV-based AGB map), the RF regression model was established for grassland AGB retrieved from the Chinese GF-2 data. The AGB estimated with RF_GF_UAV (mapping AGB from GF data based on RF model and UAV samples, similarly hereinafter) based on VIs was validated with field measurements, with an R^2^ of 0.70 and an RMSE of 78.24 g/m^2^. At the same time, in order to demonstrate whether UAV data, as a bridge between field measurements and satellite observations, can improve the estimation accuracy of satellite-scale models, the AGB retrieved directly from the GF-2 data based on RF and field measurements was also analyzed. The results show that the accuracy of RF_GF_field (mapping AGB from GF data based on RF model and field samples, similarly hereinafter) based on VIs decreased significantly; the R^2^ was decreased from 0.70 to 0.57, and the RMSE increased from 78.24 g/m^2^ to 99.38 g/m^2^ (Table 4).

**Figure 6:**
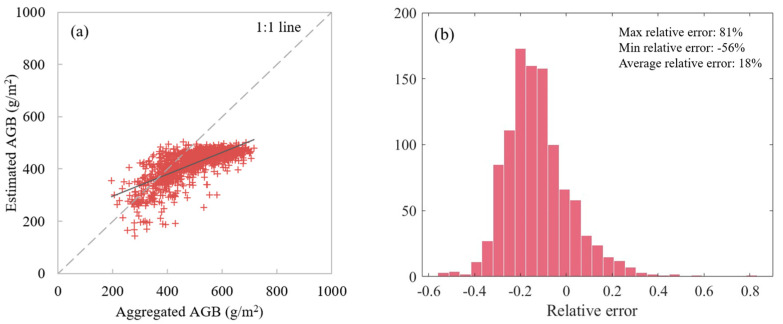
The bias between estimated AGB and aggregated AGB. (**a**) Scatterplot of estimated AGB versus aggregated AGB, (**b**) histogram of relative errors. The blue line indicates the best fit, whereas the gray line indicates the 1:1 relationship.

Due to the heterogeneity of the surface and the difference of observation resolution between different platforms, scale effects during the grassland AGB estimation based on UAV and satellite synergies cannot be ignored. The UAV-based AGB (using RF_UAV_field model) was aggregated as the relative truth value and the satellite-based AGB (using RF_GF_field model) was taken as the estimated value, and the difference between them was compared for analyzing the scale effect. As shown in Figure 6b, the scale bias reached 81%, with an average of 18%. When the resolution increases, the bias is further increased [47,48]. Therefore, it is very important to consider and introduce the scale information in the process of collaborative application of UAV and satellite data.

#### 3.2.2. AGB Estimation Based on VIs and LAI

**Figure 7:**
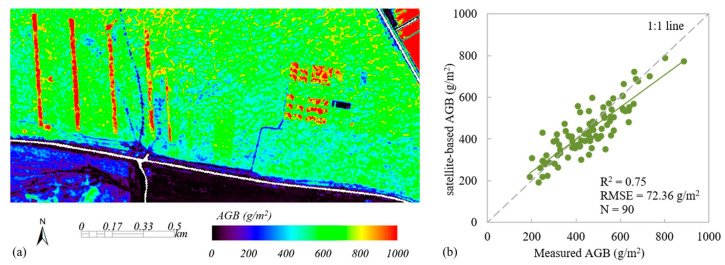
Satellite-based AGB estimation and validation. (**a**) The satellite-based AGB, (**b**) scatterplot of satellite-based AGB versus ground measurements. The green line indicates the best fit, whereas the gray line indicates the 1:1 relationship.

Firstly, based on the method described in Section 2.4.2.1, the LAI was retrieved and corrected from the Chinese GF-2 multi-spectral data. The satellite-based LAI was validated using field measurements, with R^2^ = 0.75 and RMSE = 0.63 m^2^/m^2^. Then, based on the satellite-scale AGB sample set (aggregated from UAV-based AGB map), the satellite-based LAI and Vis calculated from the GF-2 data, the RF regression model was established for estimating the grassland AGB from the Chinese GF-2 data. Finally, the performance of the AGB estimation model with and without the LAI was analyzed. Compared with RF_GF_UAV based on Vis, RF_GF_UAV based on VIs+LAI performed better; the R^2^ was increased from 0.70 to 0.75 and the RMSE was decreased from 78.24 g/m^2^ to 72.36 g/m^2^ (Figure 7b).

**Figure 8:**
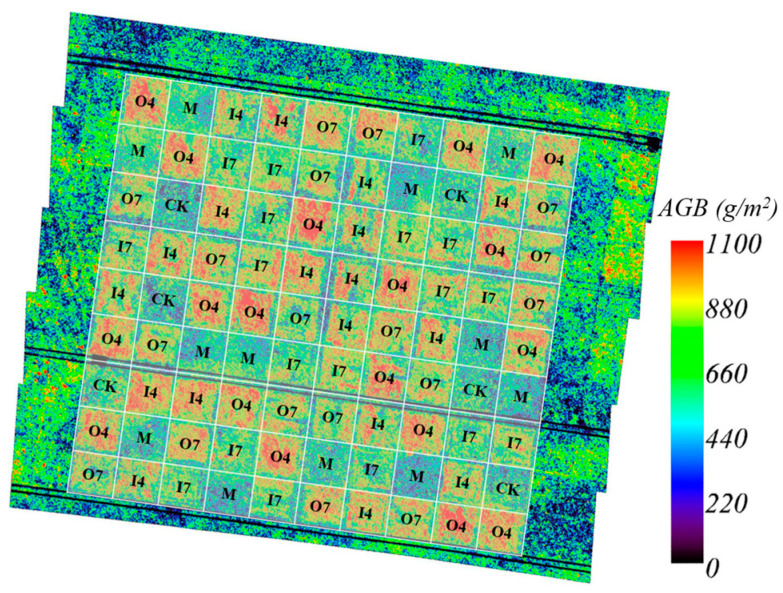
Fertilization and mowing control experiments over the 90 experimental units. CK: no control experiments were performed; M: mowing control experiments were performed; O4: organic fertilization on 10 April; O7: organic fertilization on 10 July; I4: inorganic fertilization on 10 April; I7: inorganic fertilization on 10 July.

The grassland AGB in the EP was finally mapped at 4 m resolution using the RF_GF_UAV model based on VIs+LAI. In addition, the effects of different fertilization measures on the grassland AGB were analyzed. As shown in Figure 8 and Table 5, the effect of organic fertilizer was better than that of inorganic fertilizer for grassland restoration treatment, and the effect of fertilization in April was better than that in July. Compared with the reference quadrat, the aboveground biomass of grassland increased by 45.06% after fertilization in April.

## 4. Discussion

Accurate assessment of spatio-temporal changes in grassland aboveground biomass is an essential basis for evaluating the productivity of the grassland ecosystem, monitoring grassland degradation risk and understanding the grassland ecosystem carbon cycle. Because natural grassland has extensive coverage, it is challenging to artificially obtain biomass. Moreover, it is challenging to assess grassland biomass on a large scale over long periods using only manual field surveys. It is a common way to estimate AGB estimation on a large scale by establishing relationships between measured biomass and vegetation indices calculated from satellite data. Due to the difficulty of manual ground measurement, it is impossible to obtain all the ground measurement values at the satellite pixel scale, which leads to uncertainty during AGB estimation from satellite data based on ground small quadrat data (e.g., 1 m × 1 m). As described in Section 2.4.2.1, the model established based on measured AGB and VIs calculated from GF-2 data had low accuracy, with an R^2^ of 0.57 and an RMSE of 99.38 g/m^2^. Recent advancements in UAV technology offer potential opportunities to serve as a bridge connecting satellite data and ground surveys [44]. By carrying a variety of sensors, the UAV can obtain ground samples that match the satellite pixels, especially the low- and medium-resolution satellite data.

In this study, a ground–UAV–satellite collaborative estimation strategy for grassland AGB was proposed. UAV-based AGB estimation, as the most critical link in the middle, was evaluated and analyzed first. The results demonstrate that RF had the best performance compared with SR, PLSR and BPNN, with an R^2^ of 0.72 (without LAI) or 0.80 (with LAI) and an RMSE of 83.11 g/m^2^ (without LAI) or 76.03 g/m^2^ (with LAI). The performance of the linear regression models, including SR and PLSR, was limited by the correlation between vegetation indices [72]. In contrast to these limitations of linear models, nonlinear models, including BPNN and RF, offer a different solution. The RF algorithm, an ensemble learning method based on decision trees, provided feature importance scores based on Mean Decrease Impurity (MDI). The RF model inherently incorporated a feature selection process, effectively identifying and retaining the most critical features while eliminating less important ones, which is beneficial for enhancing model interpretability and reducing overfitting. Thus, the RF was regarded as the primary model for satellite-based AGB estimation. Then, the UAV-scale AGB map was aggregated for making a satellite-based sample set. As described in Section 2.4.2.2, the AGB sample set of 1057 samples (4 m × 4 m) was achieved instead of 90 ground measurements (1 m × 1 m) by aggregating the UAV-scale AGB map. Based on this sample set, the accuracy of satellite-based AGB estimation is significantly improved, with an R^2^ of 0.70 (without LAI) and 0.75 (with LAI) and an RMSE of 78.24 g/m^2^ (without LAI) and 72.36 g/m^2^ (with LAI). This suggests that UAVs can connect the satellite observations to field measurements by providing a sufficiently large training dataset for model development and AGB estimation from satellite data.

Plant structural properties are considered significant for AGB estimation since the AGB is essentially a 3D complex plant trait [24,73,74]. At the same time, due to the saturation of optical signals, the AGB in mature grassland is easy to underestimate [20]. The introduction of structural information (e.g., canopy height) can effectively solve this problem [24,75,76]. The extraction of vegetation height using UAV LiDAR over a large area is expensive, and the flight height of UAV LiDAR has a significant impact on the capture of vegetation details [62]. At the same time, the extraction of canopy height using UAV LiDAR is still uncertain due to the influence of grass leaf droop. At the satellite scale, the estimation of vegetation canopy height of grassland also lacks the support of corresponding LiDAR data. Although some studies pointed out that satellite LiDAR data (e.g., ICESat-2, GLAS) had the potential for retrieving the forest vegetation canopy height, the uncertainty of estimations was always greater than 2 m [43,77]. These data are not suitable for grassland vegetation with a height of less than 1 m. LAI, as an important vegetation structural parameter, is also a well-known indicator for AGB modeling and can be retrieved from optical remote sensing data of different platforms. The application of LAI in grassland is mature with high accuracy and low cost, as shown in Figure 5. Grassland AGB estimation achieved higher accuracy when the LAI was introduced. For UAV-based AGB estimation, the R^2^ of models based on VIs+LAI was improved by an average of 10% and the RMSE was reduced by an average of 9% (Table 3). For satellite-based AGB estimation, RF_GF_UAV based on VIs+LAI had better performance; the R^2^ was increased from 0.70 to 0.75 and the RMSE was decreased from 78.24 g/m^2^ to 72.36 g/m^2^.

The study area was composed of multi-species communities, which were also affected by grassland restoration measures such as fertilization and mowing, so there were large differences between and within grassland species; that is, the surface heterogeneity of the study area was strong. Due to the heterogeneity of the surface and the difference in observation resolution between UAVs and satellites, scale effects must exist in the grassland AGB estimation based on UAV and satellite synergies [47]. Since the RF model is a non-parametric model, it is difficult to correct the scale bias directly. Therefore, in this study, the UAV platform was used as the intermediary, and the structural parameter LAI was introduced at the same time. Then, multi-scale information was extracted based on the NDVI and used to correct the inversion bias. Finally, the scale bias was reduced to 10%. The extraction and addition of multi-scale spatial information effectively ensure the application of the model on the heterogeneous surface. NDVI is the most widely used spectral vegetation index by ecologists and agriculturalists [9,14]. However, there is a saturation effect when NDVI is applied to densely vegetated areas, which brings uncertainty to the scale information extraction based on NDVI. Future studies could consider directly constructing an AGB-based scale correction method to solve the uncertainty caused by NDVI-based spatial correction. In addition, the synergistic use of satellite and UAV-derived data for mapping grassland AGB proposed in this study was only tested during the vegetation maturity stage, and it still needs to be optimized for different simultaneous data in different regions to improve the universality of the method.

The final AGB map of the study area also revealed that the effect of organic fertilizer was superior to that of inorganic fertilizer for grassland restoration treatment. Additionally, the effect of fertilization on 10 April was better than that on 10 July. Compared with the reference quadrat, the aboveground biomass of grassland increased by 45.06% after fertilization in April.

## 5. Conclusions

In this study, the grassland AGB was successfully estimated and mapped in the Hulunbuir meadow steppe by synergistically using Chinese GF-2 multi-spectral data and UAV hyperspectral data. The results suggest that UAVs can be used for bridging the gap between satellite observations and ground surveys. The UAV improved the performance of satellite-based AGB estimation models by providing an AGB dataset that matched the satellite pixels. This effectively reduced the effects of quadrat scale mismatch and surface spatial heterogeneity. The conclusions are as follows:

(1) RF had the best performance compared with SR, PLSR and BPNN. The grassland AGB was estimated from UAV hyperspectral data using an RF model based on VIs+LAI, with an R^2^ of 0.80 and an RMSE of 76.03 g/m^2^.
(2) Grassland AGB estimation considering the LAI achieved higher accuracy. For UAV-based AGB estimation, the R^2^ was improved by an average of 10%, and the RMSE was decreased by an average of 9%. For satellite-based AGB estimation, the R^2^ was increased from 0.70 to 0.75 and the RMSE was decreased from 78.24 g/m^2^ to 72.36 g/m^2^.
(3) During grassland AGB estimation from different platforms, the scale bias was up to 81%, with an average of 18%. By introducing spatial information during LAI inversion, the scale bias of the AGB estimation based on VIs+LAI was reduced to 10%.
(4) The final AGB map of the study area revealed that the biomass after fertilization was significantly higher than that without treatment, and the effect of organic fertilizer was better than that of inorganic fertilizer for grassland restoration treatment.

## Figures and Tables

**Table 1:** Specific description of GF-2 multi-spectral data.

Spectral Bands	Bands Range (nm)	Spatial Resolution (m)
Blue	450–520	4
Green	520–590	4
Red	630–690	4
Near-infrared	770–890	4

**Table 2:** The vegetation indices used and descriptions.

Spectral Bands	Bands Range (nm)	Spatial Resolution (m)
RVI(*i*,*j*)	$\rho_{j}/\rho_{i}$	[58]
NDVI(*i*,*j*)	$\left(\rho_{j}-\rho_{i})/(\rho_{i}+\rho_{j}\right)$	[59]
RDVI(*i*,*j*)	$(\rho_{j}-\rho_{i})/\sqrt{\left(\rho_{i}+\rho_{j}\right)}$	[60]
OSAVI(*i*,*j*)	$\frac{\left(1+0.16)\times (\rho_{j}-\rho_{i}\right)}{\left(\rho_{i}+\rho_{j}+0.16\right)}$	[61]
MSAVI(*i*,*j*)	$\frac{2\rho_{i}+1-\sqrt{{\left(2\rho_{i}+1\right)}^{2}-8(\rho_{j}-\rho_{i})}}{2}$	[62]
CI(*i*,*j*)	$\rho_{j}/\rho_{i}$ − 1	[63]

**Table 3:** Evaluation of different models with and without LAI.

Models	Based on VIs		Based on VIs+LAI	
	R^2^	RMSE	R^2^	RMSE
MLR_UAV	0.62	101.91 m^2^/m^2^	0.68	93.29 m^2^/m^2^
PLSR_UAV	0.67	87.85 m^2^/m^2^	0.72	83.61 m^2^/m^2^
BPNN_UAV	0.67	90.44 m^2^/m^2^	0.74	79.08 m^2^/m^2^
RF_UAV	0.72	83.11 m^2^/m^2^	0.80	76.03 m^2^/m^2^

**Table 4:** Comparison of statistical indicators between RF_GF_UAV and RF_GF_field models.

Models	Based on VIs	
	R^2^	RMSE
RF_GF_UAV	0.70	78.24 g/m^2^
RF_GF_field	0.57	99.38 g/m^2^

**Table 5:** Average value of AGB under different fertilization conditions.

Control Experiments	Average Value of AGB
I4	605.30 g/m^2^
O4	622.54 g/m^2^
I7	562.75 g/m^2^
O7	584.52 g/m^2^
CK	423.23 g/m^2^

## Data Availability

Data are contained within the article.
